# A Computational Model for the Release of Bioactive Molecules by the Hydrolytic Degradation of a Functionalized Polyester-Based Scaffold

**DOI:** 10.3390/pharmaceutics15030815

**Published:** 2023-03-02

**Authors:** Elisa Batoni, Amedeo Franco Bonatti, Carmelo De Maria, Kenneth Dalgarno, Raasti Naseem, Umberto Dianzani, Casimiro Luca Gigliotti, Elena Boggio, Giovanni Vozzi

**Affiliations:** 1 Research Center E. Piaggio, Department of Information Engineering, University of Pisa, 56122 Pisa, Italy; 2 School of Engineering, Newcastle University, Newcastle upon Tyne NE1 7RU, UK; 3 Department of Health Sciences, Università de Piemonte Orientale, 28100 Novara, Italy; 4 NOVAICOS s.r.l.s, Via Amico Canobio 4/6, 28100 Novara, Italy

**Keywords:** hydrolytic degradation modeling, bioactive molecules, tissue engineering, bone scaffold

## Abstract

This work presents a computational model to study the degradation behavior of polyester-based three-dimensional (3D) functionalized scaffolds for bone regeneration. As a case study, we investigated the behavior of a 3D-printed scaffold presenting a functionalized surface with ICOS-Fc, a bioactive protein able to stimulate bone regeneration and healing, inhibiting osteoclast activity. The aim of the model was to optimize the scaffold design to control its degradation and thus the release of grafted protein over time and space. Two different scenarios were considered: (i) a scaffold without macroporosity presenting a functionalized external surface; and (ii) a scaffold presenting an internal functionalized macroporous architecture with open channels to locally deliver the degradation products.

## 1. Introduction

The standard treatments for bone defects resulting from trauma or degenerative conditions rely on metal fixation which may lead to problems due to poor osteoconductive potential or may require a second surgery if not biodegradable [1]. Bone tissue engineering (BTE) offers a promising approach to bone repair by using scaffolds, cells, and biomolecules to promote the formation of the new tissue. The scaffold can be fabricated in different materials such as bioresorbable polymers [2]. Among these, aliphatic polyesters such as polyglycolic acid (PGA), poly-lactic acid (PLA), their copolymer poly(lactic-co-glycolic acid) acid (PLGA), and blends have been considered for bone regeneration since these materials can undergo degradation in vivo and are approved by the U.S. Food and Drug Administration [3,4]. In addition, the PLA stereoisomeric forms such as poly(L-lactic) acid (PLLA), poly(D-lactic) acid (PDLA), and also poly(D,L)-lactic acid) (PDLLA) have been extensively used for BTE applications [4,5]. These polymers can also be chemically modified in different ways such as by grafting. In fact, bioactive molecules such as proteins can be grafted on the polymeric surface for a controlled delivery because of the degradation of the polymer matrix [6,7].

Specifically, the in vivo degradation starts with the hydrolysis of the polymer, consisting in the scission of the polymeric ester backbone into monomers (e.g., lactic acid (LA) or glycolic acid (GA) monomers), which are then metabolized and safely eliminated from the body [4]. The hydrolysis process is mainly divided into four stages: (i) firstly, the water molecules diffuse through the polymer matrix; (ii) then, the ester bonds of the polymer backbone break, and the hydrolysis reaction is autocatalyzed by the increasing number of the acid carboxylic end groups [4]; (iii) as the molecular weight of the polymer decreases, the integrity of the polymer is no longer held and the mass loss begins; and (iv) then, the erosion starts resulting in the further cleavage of fragments, which are soluble in the surrounding medium [4,8]. The autocatalytic reaction is caused by the degradation products, which may slowly diffuse in the large-sized samples and accumulate inside the polymer matrix, accelerating the degradation process [9].

It is worth highlighting the difference between the two terms, degradation and erosion: while the first refers to the chemical phenomena which leads to the hydrolytic bond cleavage to form degradation products, i.e., monomers and oligomers, the second is a broader term which indicates the physical phenomena of the degradation products’ dissolution and diffusion in the environment. The erosion of the polymeric matrix can proceed through two different physical mechanisms: (i) surface erosion, which is limited to the material surface and proceeds inward; and (ii) bulk erosion, which occurs throughout the material volume [3]. The first usually occurs when the rate of the water diffusion is slower than the polymer degradation, while in the second the water diffuses at a faster rate than that at which the polymer is being hydrolyzed [10]. In the literature, several mathematical models have been proposed for the prediction of polymer hydrolysis, which is useful to predict the release of the degradation products in different conditions and designs [10].

### 1.1. Mathematical Models for the Hydrolytic Degradation

According to the literature, hydrolytic degradation models are divided into three different types: phenomenological, probabilistic, and empirical [11]. The first is based on reaction–diffusion equations, where the reaction accounts for the hydrolysis, and the diffusive part for the diffusion of the degradation products, while the second and the third are based respectively on probability distributions or empirical data to describe the changes in the polymer properties [11]. Among these, phenomenological models are applied to a wide variety of polymers, such as polyesters [9,11].

Some models take into consideration the diffusion of the water molecules towards the polymer matrix, but not the autocatalytic effect. For instance, Shokley et al. [12] presented a phenomenological model to account for water diffusion, the hydrolytic process (without autocatalysis), and the formation and diffusion of the monomers out of the polymer matrix. The model parameters were calibrated by iteratively fitting the experimental data for PLGA samples.

On the contrary, Vieira et al. described the hydrolytic process with a first-order equation without considering the water diffusion, assumed to be faster than the hydrolysis reaction [13,14]. In fact, most studies ignore the diffusion of the aqueous medium assuming the polymer is fully saturated because the sample size is small and/or the water diffusion occurs faster than the other processes. Instead, more attention is given to the autocatalytic effect which is, in turn, a key factor in the degradation of PLA and PGA [15]. For example, Antheunis et al. [16] presented a mathematical model considering the hydrolysis equations based on only the autocatalysis, which depends on the ester bonds and the acid concentrations. On the contrary, Wang et al. [17] introduced a phenomenological reaction–diffusion model accounting for the autocatalyzed and non-catalyzed hydrolysis, as well as the monomer diffusion. The parameters were calibrated and compared with the experimental data of the PDLLA samples. This model was then used in several studies to predict and validate the degradation of various polyesters. Shine et al. [18] implemented Wang’s model to evaluate the degradation of a bioresorbable polymeric stent made in PLA, PLLA, and PLGA. Instead, Shirazi et al. [9] used the same model to study the degradation behavior of PLGA scaffolds with three different architectures. The mechanical behavior was also investigated by coupling the degradation with a mechanical model to relate the molecular weight with the Young’s modulus. Furthermore, Heljack et al. [19] integrated Wang’s model to predict the effect of environmental conditions, such as the presence of a flowing medium, and the frequency at which the surrounding medium is replaced, on the hydrolytic degradation.

### 1.2. Purpose of the Study

This study presents a computational model for the hydrolytic degradation study of a 3D polyester-based scaffold that is used to treat periprosthetic osteoporotic fractures in long bones (e.g., femur, tibia…). The scaffold has a “C-block” shape to adapt to the bone curvature and is supposed to be kept in contact with the periosteum by fixation mechanisms such as fixation plates, screws, and/or cerclage wires.

In recent years, additive manufacturing (AM) technologies, such as fused deposition modeling (FDM), are gaining more interest in scaffold fabrication techniques due to their high reproducibility, accuracy, and repeatability. For instance, FDM could be useful in BTE because of its capability to produce complex parts and custom shapes at minimal operational costs. This can be used to fabricate scaffolds by the layer-by-layer deposition of a wide range of thermoplastic polymers such as polyesters (e.g., PLLA, PLGA, or PDLLA) [2]. Considering the geometrical complexity of bone scaffolds, as well as the need to fabricate it in an accurate and repeatable manner, the modeling study was implemented on scaffolds 3D printed by FDM with PLLA-based filaments. The 3D-printed scaffolds are then functionalized with ICOS-Fc on its external surface, a bioactive molecule whose signals stimulate bone regeneration and wound healing inhibition osteoclasts activity [20], to enhance its biological properties and healing potential.

Two different scenarios for the functionalization were investigated: (i) ICOS-Fc grafted externally on the surface placed in contact with the bone; and (ii) a scaffold presenting macropores where ICOS-Fc is internally grafted. In the first scenario, ICOS-Fc will mainly act as a signal molecule on the cells as long as the surface is present, whereas in the second one, the bioactive molecules are grafted on the macroporous surface and delivered through the open channels as the surface degrades.

The macroscopic porosity will be permeated by body fluids or eventually infilled with a hydrogel (e.g., collagen), where the degradation products will diffuse and be delivered from the open channels towards the fracture. Since the biomolecules are grafted on the polymeric surface, their release from the opening channels will be based on the scaffold hydrolytic degradation and the diffusion of the degradation products (i.e., the monomers and oligomers) from the internal macropores filled with the hydrogel. In this scenario, channels in contact with the bone can be closed to guide the bioactive protein delivery over space (direct the release towards a specific bone area). In the following sections, the scaffold is referred to as a “3D complex scaffold”.

To have a controlled degradation and/or release of the molecules, the scaffold design and macroporosity can be optimized according to the results given by the presented computational model. The simulation framework is divided into the following steps: (i) firstly, a mathematical model of the hydrolytic degradation for PLLA-based materials was found in the literature, and implemented in the COMSOL Multiphysics^®^ Finite Element (FE) simulation platform; (ii) the model parameters were calibrated and validated through experimental data of in vitro degradation of PLLA pellets; (iii) the model was implemented on the externally grafted 3D complex scaffold (first scenario), and on the one presenting an internally grafted macroporosity (second scenario), with different delivery conditions (i.e., all and only four central opening channels); (iv) finally, the optimal design was selected by evaluating the degradation of the contact surface and the release of the degradation products, over time and space.

## 2. Materials and Methods

### 2.1. Mathematical Model for the Hydrolytic Degradation

Among the mathematical models found in the literature, Wang’s model [17] was used in this study. Although this is a simplified model, it captures the key features of the hydrolysis process, and the authors demonstrated its valid predictions with experimental observations [17]. Furthermore, the model was implemented in several studies demonstrating good accordance with the experimental degradation data of different polyester-based samples, i.e., PLLA, PLGA, and PDLLA [9,17,18].

The model is based on some assumptions which were also hypothesized in this study: (i) the size and the distribution of the polymer chains and the hydrolysis products are ignored; (ii) the water penetration into the polymer matrix is assumed to be faster than the other kinetics, thus, the molecules are assumed to be abundant (and thus not considered in the equations); (iii) the degree of crystallinity of the polymer does not change during the degradation [17].

The mathematical model is based on three main equations: (i) a reaction equation representing the breakage of ester end groups in the polymer matrix (Equation (1)); (ii) a reaction–diffusion equation to capture the time and space distribution of the degradation products, i.e., the monomer and concentration (Equation (2)); and (iii) an expression for the monomer diffusion coefficient which is dependent on the time-varying ester end groups and monomer concentration, which takes into account the increasing porosity during the degradation (Equation (3)) [17]. Thus, the three main variables are the molar concentration of the ester end groups, indicated as Ce[molm3 ], and the molar concentration of the monomers, i.e., Cm[molm3 ], representing the degradation products of the hydrolytic reaction [17]. The equations are reported and described below:

- Ester groups concentration: The reaction rate of the ester groups considers two effects occurring during the degradation, i.e., the non-catalysis and autocatalysis of the polymer chains, by using two different constants, k1[1s] and k2[m3/mols], respectively (Equation (1)). In the autocatalytic term, the exponent β of $C_{m}$ is used to account for dissociation of the acid ester end groups and to capture the non-linearity of the reaction [9,17]. Several studies set β equal to 0.5 [9,17,18].
  (1) $$\frac{dC_{e}}{dt}=-\left(k_{1}C_{e}+k_{2}C_{e}C_{m}{}^{\beta }\right),$$
- Monomer concentration: While the ester end groups remain inside the polymer matrix without diffusing, the monomers diffuse throughout the polymer as the chains break. As a result, the ester group’s reaction, and thus, the monomer production, is accompanied by a diffusion term given by the divergence of the gradient of $C_{m}$ (Equation (2)) [9,17].
  (2) $$\frac{dC_{m}}{dt}=k_{1}C_{e}+k_{2}C_{e}C_{m}{}^{\beta }+\nabla \cdot D\nabla C_{m},$$
- Monomer diffusion coefficient: The monomer diffusion coefficient in the polymer matrix increases as the products diffuse out leaving void pores in the polymer matrix. Thus, the diffusivity, i.e., D [m2s], depends on the intrinsic diffusion coefficient, $D_{0}$, which is the diffusivity of the monomers before the degradation occurs, and the porosity $p=\left(1-\frac{C_{e}+C_{m}\;}{C_{e0}}\right)$, through a linear relation, where α was kept equal to 4.5. The porosity is defined as a function of $C_{m}$ and $C_{e}$, where $C_{e0}$ indicates the initial concentration of the ester groups (Equation (3)) [9,17].
  (3) $$D=D_{0}\left[1+\alpha \left(1-\frac{C_{e}+C_{m}}{C_{e0}}\right)\right],$$

### 2.2. Model Implementation in COMSOL Multiphysics^®^

The previous equations were implemented in the FE software platform COMSOL Multiphysics^®^. The model setup was validated by simulating different scenarios of polyester-based samples reported in the literature and comparing the simulation results with their experimental data [21].

In detail, as suggested by the literature [9], a simplified model, i.e., 1D axisymmetric, was implemented considering the symmetry of the geometry and the boundary conditions. Regarding the model setup, the “Transport of Diluted Species” physics was chosen with two dependent variables, $C_{e}$ and $C_{m}$, referring to the ester group and the monomer concentrations. As previously stated, the water molecules are not considered as it was assumed to be abundant in the polymer.

In the domain properties, the diffusion coefficient of the ester groups was set to 0 m2s (no diffusion occurs), while the monomer diffusivity, i.e., D, was described as reported in Equation (3).

A reaction boundary was applied to the whole domain to account for the reaction and the production of respectively, the ester groups and the monomers, implemented as reported in Equations (1) and (2). In addition, a concentration equal to 0 molm3 was set as a boundary condition on the interface between the polymer and the medium, hypothesizing that the monomers reaching the surface are immediately washed out through the medium [9]. The parameters of each equation were found in the literature and are reported in Table 1 [9,17].

### 2.3. Parameter Calibration for PLLA

The in vitro degradation of PLLA was assessed via gel permeation chromatography (GPC) of rod samples (n = 3; dimensions of 1.6 mm in diameter and 6 mm height) at 50 °C in phosphate-buffered saline (PBS) 1X (phosphate-buffered saline, Sigma-Aldrich) for 2 months. Degradation tests on PLLA pellets were performed at 50 °C to accelerate the polymer degradation due to the increase in chain and mobility [22,23,24].

Since the hydrolytic degradation is strongly affected by the temperature [22,23], the parameters (i.e., $k_{1},k_{2},D_{0}$) are temperature dependent, thus, the previous ones were not applied since they were calibrated and validated at 37 °C in PBS [9,17,18].

According to the literature, the Arrhenius relation can be applied to determine the model parameters (e.g., $k_{1},k_{2},D_{0}$) at different temperatures [23,24]. The Arrhenius relation comprises two constants: the activation energy, $E_{a}\;\left[J\right]$, and a pre-exponential factor, A [25].

The activation energies for each parameter were taken from the literature [24], whereas the pre-exponential factors were computed with the Arrhenius equation using the parameters validated at 37 °C for the degradation of PLA plates, and the respective activation energies [26].

**Figure 1:**
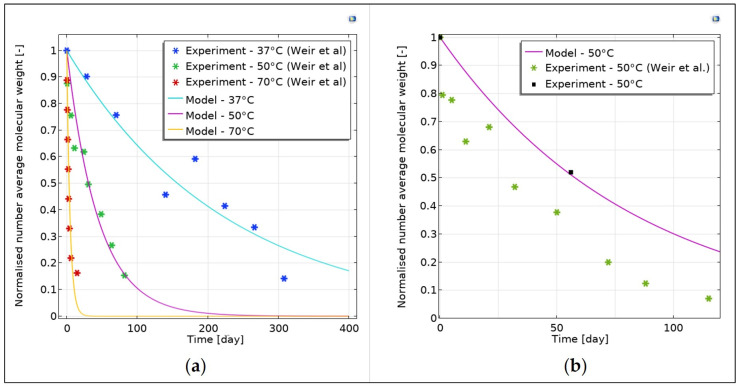
Parameter calibration for PLLA. (**a**) Validation of the Arrhenius constants with the experimental data of PLLA samples provided by Weir et al. [27]; (**b**) calibration and validation for PLLA rods at 50 °C in PBS.

The values of the pre-exponential factors were validated by comparing the experimental degradation data of PLLA samples in PBS at 50 °C and 70 °C with the simulated results [27] (Figure 1a).

Then, the parameters 50 °C were used to simulate the in vitro degradation of the PLLA rods and the number average molecular weight was compared with the experimental values. The parameters were slightly varied one at a time until the percentage error between the experimental and the simulated values was negligible (Figure 1b). The Arrhenius relation was then re-applied to estimate the parameters for the in vitro degradation of the PLLA rods at 37 °C.

### 2.4. Model Implementation on a 3D Complex Scaffold

The 3D complex scaffolds present a “C-shape” geometry, with dimensions of 50 mm height, 40 mm width, and 10 mm/20 mm thick. While in the first scenario, the scaffold has no porosity, in the second scenario scaffolds present an interconnected macroporous architecture with open channels towards the external environment. Specifically, the macropores have an ellipsoidal shape (1.5 mm, 2 mm, and 1.5 mm) connected to each other with cylindrical channels (2 mm diameter, 2 mm length). Open channels (2 mm diameter, 1 mm length) are present on the lateral side to fill the scaffold with hydrogel. For each scenario, 2 scaffolds were considered differing in thickness, i.e., 10 mm and 20 mm thick. A double order of interconnected macropores was created in the 20 mm thick model. The scaffolds are supposed to be fabricated with a standard FDM printer with PLLA-based filaments [28].

**Figure 2:**
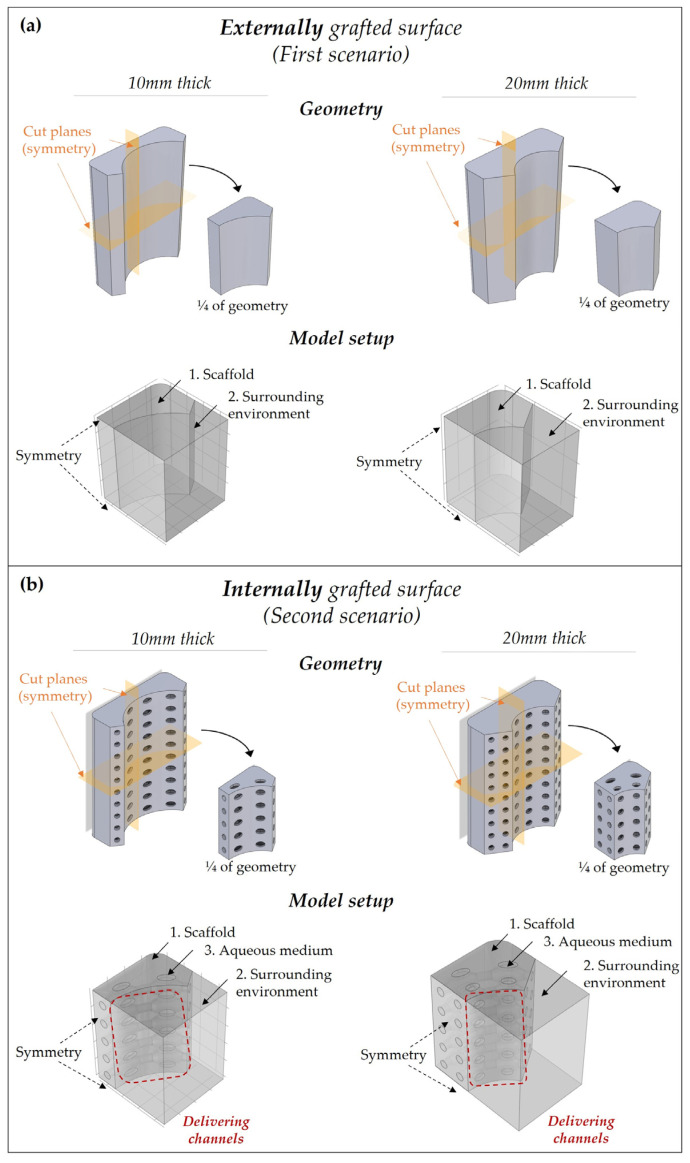
Model implantation on a 3D complex scaffold in the two different scenarios: (**a**) externally grafted and (**b**) internally grafted with ICOS-Fc. Geometry: due to the presence of two mid-planes, 1/4 of the geometry was investigated. Model setup—domains: (1) polyester-based scaffold, (2) surrounding environment (i.e., periosteum); and (3) aqueous medium within the macroporous architecture. Boundary conditions: symmetry conditions were placed on the mid-plane cutting surfaces; the concentration boundary was placed on the interface between each domain as a continuity condition; and in the second scenario, no flux conditions were set on the channel surfaces except for those open for delivery.

Since the scaffolds present two mid-planes, the study was conducted on ¼ of the total geometry to reduce the computational cost and time (Figure 2a,b).

As in the simplified model, the “Transport of Diluted Species” physics was used but two/three domains were now considered: (i) polyester-based scaffold; (ii) the surrounding domain (which should represent the periosteum of the bone fracture); and (iii) specifically for the second scenario, an aqueous medium inside the porous architecture (Figure 2a,b).

Regarding the boundary conditions, a concentration boundary was set to the interface between the scaffold, the medium, and the surrounding environment to ensure the continuity of the concentrations between the different domains. The diffusivity of the degradation products in the aqueous medium and the surrounding environment was supposed to be equal to 10−9 m2s, which agreed with values found in the literature for bioactive molecules [29,30].

In the second scenario, no flux conditions were placed on the channels which were not left open for the delivery, like the lateral ones. To evaluate the optimal condition to have a time/space-controlled release of the bioactive molecules, two different conditions for the open channels were considered, respectively: all channels open, or only four central open channels (specifically, one open channel for the ¼ geometry). In the second case, no flux condition was placed in all delivering pores except for the central ones.

In all models, symmetry conditions were applied to the mid-plane cutting surfaces. A time-dependent study was chosen to evaluate the behavior of the degradation and the release of the bioactive molecules over 420 days. The parameters of the hydrolysis equations were set according to the calibration step reported in Section 2.2.

## 3. Results

### 3.1. Model Calibration and Validation

The activation energy and the pre-exponential factors fitted well with the experimental data provided by Weir et al. [27] of PLLA plates at different temperatures (e.g., 37 °C–50 °C–70 °C) as shown in Figure 1b.

Regarding the PLLA pellets, the fitting was performed with experimental data obtained by degradation at 50 °C of PLLA pellets (n = 3) in PBS 1X solution. The molecular weight of each sample was measured by GPC at the beginning of the test (t = 0 days) and after almost 2 months (t = 56 days). The hydrolytic degradation parameters were then adjusted to fit the curve with the molecular weight measured at the last time point, giving the following values: 2·10−3 1day for $k_{1}$, 1.2·10−7 m3/molday for $k_{2}$, and 6.5·10−12 m2day for $D_{0}$. The percentage error at 56 days was around 2%, demonstrating a good fitting of the curve. Furthermore, the estimated values are in line with those found in the literature [18], thus validating the obtained results.

### 3.2. Degradation Behaviour of the 3D Complex Scaffold

**Figure 3:**
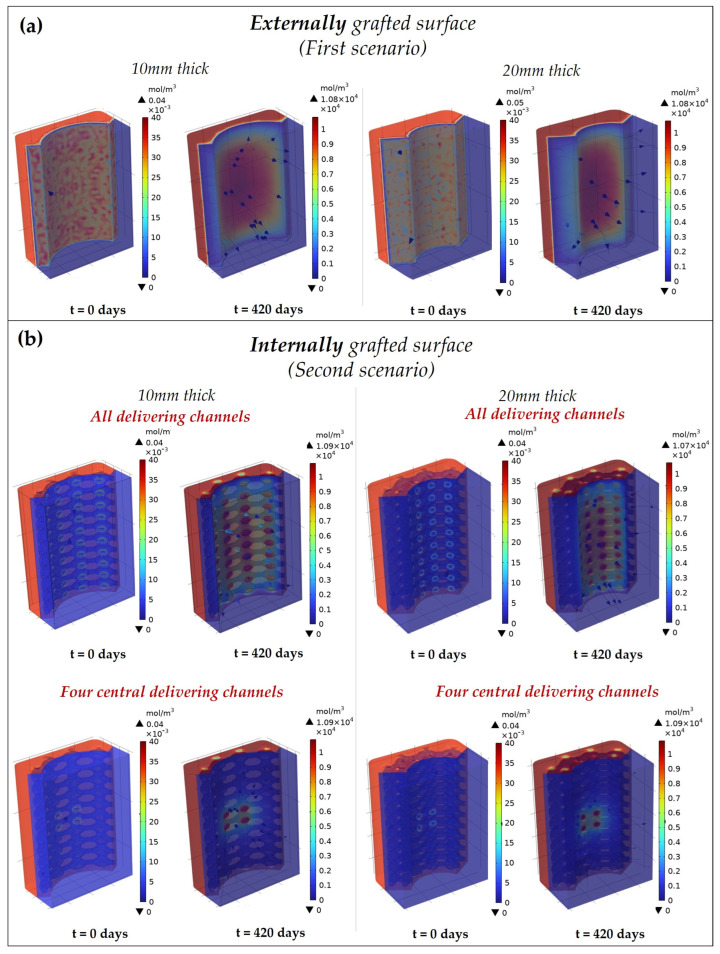
Degradation behavior of the 3D complex scaffold in the two scenarios: (**a**) externally (first scenario) and (**b**) internally (second scenario) grafted with ICOS-Fc. The second scenario is shown both with all and only four central delivery channels open. The spatial distribution of the grafted protein in the different domains (represented by the monomer concentration) is reported at 2 different time points: t = 0 days, at the beginning the hydrolysis is not yet started and the protein is completely grafted on the scaffold; at t = 420 days (end time point) the products diffuse towards the periosteum from the external or internal grafted surface. The flow direction of the degradation products is highlighted by the streamlines.

The degradation behavior of the scaffolds was evaluated considering the time–space variation of the monomer concentration (representing the degradation products) on the surfaces theoretically grafted with the bioactive protein. For each scenario, the spatial distribution of the products is represented in Figure 3a,b, at two different time points: t=0 days (start point) and t=420 days (end point).

In each scenario, at the beginning the monomers were homogeneously distributed in the volume; then, as the hydrolysis reaction occurs, the products left the polymeric matrix and diffused into the surrounding environment. In the second scenario, the products initially diffuse in the aqueous medium within the macropores, then towards the surrounding environment through the open channels (as highlighted by the streamlines in Figure 3b).

While in the first scenario the products diffuse equally from the grafted surface, in the second one the release is directed towards a specific area by the open channels. The localized release is evident when only four central channels are open, where the products locally diffuse from the internal channels towards the bone.

Considering the thickness, the grafted protein is similarly distributed in the space for each scenario, but the concentration is greater in the 20 mm thick scaffold since more material is present.

### 3.3. Release Kinetic of the Bioactive Molecules

**Figure 4:**
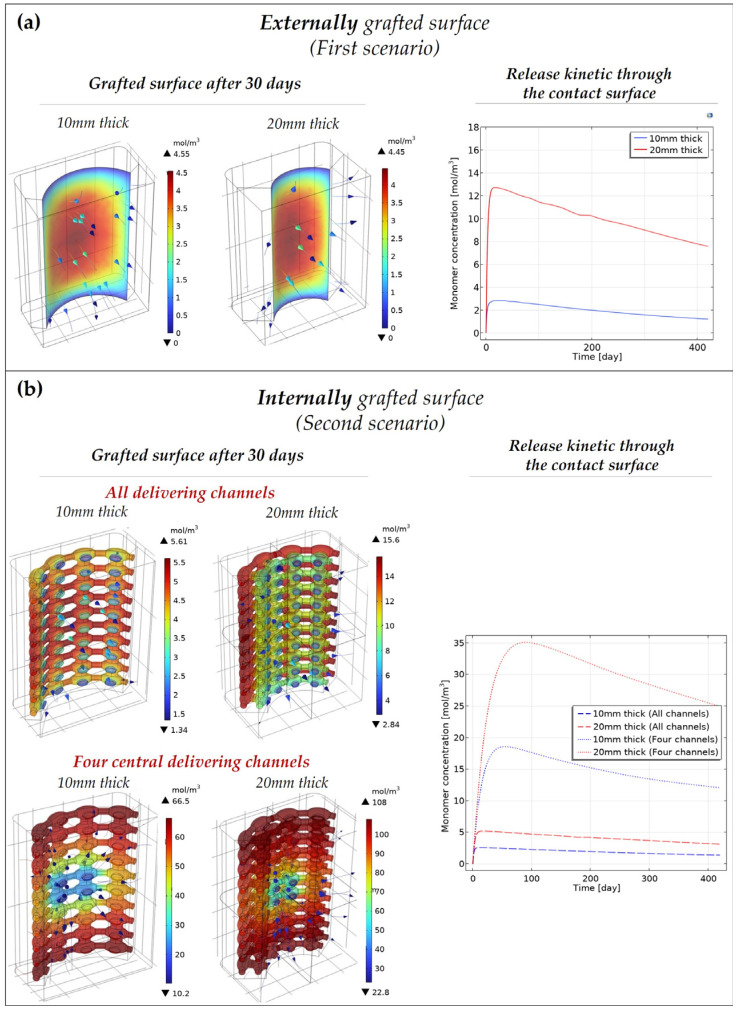
Release kinetic of the bioactive molecules through the contact surfaces (i.e., external grafted surface in the first scenario (**a**) and the open delivering channels in the second scenario (**b**)) of the scaffold. Depicted on the left is the degradation behavior of the grafted surface—(**a**) internal (first scenario) and (**b**) external (second scenario)—after 30 days. The release kinetic of the degradation products through the contact surfaces is reported on the right.

The amount of grafted protein released through the contact surface (external surface in the first scenario, and open channels in the second scenario) is reported in Figure 4a,b. While in the bulk geometry the protein slowly diffuses from the contact surface, the release kinetic is faster in the porous geometry (second scenario), particularly when only four channels are open. In each scenario, the monomer concentration released on the contact surface has an initial burst and then slowly decreases. This might be due to the monomer diffusion in the surrounding environment which is faster than the hydrolytic production and diffusion through the degrading polymeric matrix. In fact, the monomer release depends on three different phenomena (i.e., non-catalytic reaction, autocatalytic reaction, and monomer diffusion in the polymeric matrix) with a specific characteristic time representing how fast the process will proceed. Considering the 20 mm thick scaffold, the polymeric matrix diffusion time is around $8\cdot 10^{12}\;s\;\left(t=\frac{thickness^{2}}{D}\right)$ against $4\cdot 10^{7}\;s\;\left(t=\frac{1}{k_{1}}\right)$ and $6\cdot 10^{9}\;s\;\left(t=\frac{C_{e0}}{k_{2}}\right)$ for the non-catalytic and autocatalytic reactions, respectively. Thus, the diffusion of the grafted protein is limited by the diffusion of the degradation products in the polymeric matrix, which is due to the low matrix diffusion coefficient (D≅10−17 m2s at 37 °C).

Comparing the 10 mm and 20 mm thick scaffold for each scenario, the number of bioactive molecules released over time is higher in the second since more material is present.

The porous geometry (second scenario) is preferred for a controllable release of the protein over space. In fact, to localize the action of the released protein on a particular area, the case with only four central channels open would be the best solution. However, if the aim is ensuring the contact between the bone and the active surface as much as possible over time, and not to localize the action of the active protein, the first scenario will be the optimal case, as the erosion of the contact surface (i.e., the diffusion of the degradation products) is slower than in the porous geometry.

## 4. Discussion

The model parameters were estimated considering the degradation experiments of PLLA. Data were firstly obtained from PLLA degradation tests at 50 °C in PBS 1X solution, an approach usually performed to accelerate the polymer degradation (specifically for those degrading in more than 3 years) since testing for such a long period would be time-consuming. It is worth highlighting that elevated temperature may alter the degradation mechanism, making the data obtained at elevated temperature irrelevant to degradation at 37 °C [24]. However, this phenomenon was investigated by Weir et al. [27], who carried out degradation experiments on PLLA samples at 37 °C, 50 °C, and 70 °C, concluding that degradation proceeds in a similar way at the elevated temperatures to that observed at 37 °C in vitro. Thus, in this study, the hydrolytic degradation parameters were estimated at elevated temperature and then, by using the Arrhenius relationship, values at 37 °C were extrapolated. Even though the parameters for the Arrhenius equation pertained to experimental data provided by the degradation of 2D plates [25,26], these are still valid for this study since the parameters mainly depend on the material composition (i.e., copolymer composition, crystallinity etc.) but not on its geometry and shape [5].

Although the presented model can be used to predict the hydrolytic degradation behavior of polyester-based scaffolds, there are some limitations that will be further studied in the future. For instance, the presence of grafted protein may affect the degradation behavior which should be investigated with more in vitro studies. Another important point is the effect of the degradation behavior on the scaffold mechanical properties: in fact, as the molecular weight decreases, the scaffold may become more brittle and susceptible to higher deformation if stress is applied [5,9]. However, the model can be upgraded with other equations to relate the changes in the elastic modulus with the molecular weight. Another crucial point is the diffusion coefficient of the degradation products, which in this case is supposed to be equal to that of the bioactive protein in water-based solutions. However, since the scaffold is meant to be in contact with the periosteum, the products may diffuse slower due to the high-density tissue. This could also be modeled by changing the diffusion coefficient with that of the target surrounding tissue.

Furthermore, researchers reported that polyesters can exhibit enzymatic degradation when in a biological environment, such as a patient’s body. Since enzymes are large molecules, no diffusion occurs towards the crystalline regions, but they promote the surface erosion of the polymer. For instance, a change in the degradation rate of PLLA was observed due to the presence of enzymes such as pronase, proteinase K, and bromelain [31]. Additionally, an increasing degradation rate of PLLA cultured with microorganisms compared with degradation in an abiotic environment has been reported [32]. However, passive hydrolysis is stated to be the most important degradation mode in almost all biodegradable materials, especially in synthetic polymers [13].

A role in scaffold degradation may be played by osteoclast activity through the release of their enzymatic repertoire including acid phosphatase and several proteases, such as cathepsins and metalloproteinases. Therefore, proteases might be involved in enzymatic degradation, whereas phosphate ion release by acid phosphatase-mediated hydrolysis of the bone calcium phosphate has been shown to enhance PLGA hydrolysis [33]. ICOS-Fc grafting in the scaffold would provide a selective anti-resorption activity by inhibiting osteoclast activity, which may favor bone repair and delay scaffold degradation. It would be an optimal strategy to localize ICOS-Fc at the site of active erosion, avoiding possible systemic side effects [20,33]. Another crucial point is the role of ICOS-Fc in bone healing: if ICOS-Fc is meant to act as a signaling molecule, the optimal scenario would be the first where the polymeric matrix is directly in contact with the periosteum. Instead, in the porous condition, the grafted protein will not act until it reaches the bone interface through the opening channels, which is limited by the low diffusivity of the polymeric matrix. However, it is unclear whether their diffusion, and thus controllable release, can be beneficial for healing. Thus, a combination of diffusion and signaling might be a good alternative, grafting the bioactive molecule onto both the internal macroporous surface and the external contact surface of the porous scaffold.

It is important to highlight that the nature of polyester will influence the degradation behavior of the scaffold in terms of the rate of hydrolytic degradation. In fact, according to the literature, PLLA exhibits a slower degradation rate compared with PDLLA and PLGA due to the presence of higher amounts of amorphous regions [4,5]. Thus, according to the polyester’s nature, some parameters of the hydrolytic degradation model must be changed accordingly. As reported by Shine et al. [18], the most influenced parameters are the non-catalytic ($k_{1}$) and autocatalytic ($k_{2}$) rates, as well as the diffusion coefficient ($D_{0}$), representing the diffusion of the degradation products within the polymeric matrix. For instance, since PLGA degrades faster, these parameters exhibit higher values than those estimated for PLLA, meaning that more degradation products are produced and diffuse faster within the polymeric matrix.

Regarding the scaffold fabrication technique, it is important to highlight that, even though the model was implemented on scaffolds fabricated by FDM, the model setup is still valid for other fabrication techniques since the parameter values of the hydrolytic degradation modeling mainly depend on the material composition [4].

## 5. Conclusions

In summary, the presented study demonstrated a computational model for evaluating the degradation behavior of a polyester-based scaffold functionalized with the ICOS-Fc protein. Different scaffold designs were evaluated to predict the release of the degradation products from the scaffold towards the periosteum in space and time. The model results can be used to optimize the scaffold design, its macroporous architecture (e.g., all, only the four central, or zero open channels), and geometry (i.e., 10 mm or 20 mm thick) to obtain a controlled release of the degradation products in space and time.

As a future development, the model will also account for the influence of the environmental conditions (i.e., the presence of enzymes and pH) on the degradation behavior of the scaffold by adding specific reaction equations on the polyester-based scaffold domain.

## Figures and Tables

**Table 1:** Parameters used for the model implemented in COMSOL Multiphysics^®^ for PLGA.

Parameter	Value	Description
$C_{e0}$	17,300 molm3	Initial concentration of the ester end groups [17]
$k_{1}$	0.002 1day	Non-catalytic reaction rate for PLGA [9]
$k_{2}$	0.002 m3/molday	Autocatalytic reaction rate for PLGA [9]
$D_{0}$	10−12 m2day	Initial diffusion coefficient of the monomers for PLGA [9]

## Data Availability

Not applicable.

## Abbreviations

**Abbreviation**	**Definition**
BTE	Bone tissue engineering
PGA	Polyglycolic acid
PLA	Polylactic acid
PLGA	Poly (lactic-co-glycolic acid)
PLLA	poly(L-lactic) acid
PDLA	poly(D-lactic) acid
PDLLA	poly(D,L)-lactic acid
LA	Lactic acid
GA	Glycolic acid
AM	Additive manufacturing
FDM	Fused deposition modeling
GPC	Gel permeation chromatography
